# Dual and selective inhibitors of pteridine reductase 1 (PTR1) and dihydrofolate reductase-thymidylate synthase (DHFR-TS) from *Leishmania chagasi*

**DOI:** 10.1080/14756366.2019.1651311

**Published:** 2019-08-14

**Authors:** Bárbara Velame Ferreira Teixeira, André Lacerda Braga Teles, Suellen Gonçalves da Silva, Camila Carane Bitencourt Brito, Humberto Fonseca de Freitas, Acássia Benjamim Leal Pires, Thamires Quadros Froes, Marcelo Santos Castilho

**Affiliations:** a Programa de Pós-Graduação em Farmácia, Universidade Federal da Bahia, Salvador, BA, Brazil;; b Programa de Pós-Graduação em Biotecnologia, Universidade Estadual de Feira de Santana, Feira de Santana, BA, Brazil;; c Programa de Pós-Graduação em Ciências Farmacêuticas, Universidade Estadual da Bahia, Salvador, BA, Brazil;; d Departamento de Ciências da Vida, Universidade do Estado da Bahia, Salvador, BA, Brazil

**Keywords:** Dual inhibitors, pteridine reductase 1, dihydrofolate reductase-thymidylate synthase, *Leishmania chagasi*, selective inhibitors

## Abstract

Leishmaniasis is a tropical disease found in more than 90 countries. The drugs available to treat this disease have nonspecific action and high toxicity. In order to develop novel therapeutic alternatives to fight this ailment, pteridine reductase 1 (PTR1) and dihydrofolate reductase-thymidylate synthase (DHF-TS) have been targeted, once *Leishmania* is auxotrophic for folates. Although PTR1 and DHFR-TS from other protozoan parasites have been studied, their homologs in *Leishmania chagasi* have been poorly characterized. Hence, this work describes the optimal conditions to express the recombinant *Lc*PTR1 and *Lc*DHFR-TS enzymes, as well as balanced assay conditions for screening. Last but not the least, we show that 2,4 diaminopyrimidine derivatives are low-micromolar competitive inhibitors of both enzymes (*Lc*PTR1 Ki = 1.50–2.30 µM and *Lc*DHFR Ki = 0.28–3.00 µM) with poor selectivity index. On the other hand, compound **5** (2,4-diaminoquinazoline derivative) is a selective *Lc*PTR1 inhibitor (Ki = 0.47 µM, selectivity index = 20).

## Introduction

Leishmaniasis is an infectious disease caused by several species of the genus *Leishmania*, which are responsible for its different epidemiological and clinical characteristics^1^. Visceral leishmaniasis (VL), the most severe clinical form of leishmaniasis, affects 300,000 people worldwide, 90% of whom would die if no treatment is provided^2^. The available drugs to fight this disease have been in the market for more than 40 years and for that reason, resistant strains have been selected^3^. On top of that, N-methyl glucamine (Glucantime^®^), the drug of choice to treat patients with leishmaniasis, may cause nephrotoxicity, anorexia, abdominal pain, lethargy and cardiotoxicity^4^ and the second-choice drug (amphotericin B) shows cardiac and nephron toxicity^5^. Although two new drug candidates to treat VL entered clinical tests in 2017^6^, the search for new safe, effective, oral, short-course leishmanicidal agents continues to be a priority. In order to achieve this goal, several efforts have been made to develop compounds that inhibit carbonic anhydrase^7–9^, arginase^10^, or enzymes that protect the parasite from the oxidative stress^11^^,^^12^.

Trypanosomatids dependence on host-synthesized purine and pteridines has long been targeted to fight Chagas disease^13^, human African trypanosomiasis^14^, and leishmaniasis^15^^,^^16^. Among the enzymes that play a key role in the salvage and folate pathway, dihydrofolate reductase is the macromolecular target of several drugs, including those available to fight malaria^17^. However, the selection of pyrimethamine-resistant *Plasmodium falciparum* strains underscores the risks of blocking a single target from this pathway^18^. Single point mutations^19^, as well as biochemical pathway plasticity^17^^,^^20^, can significantly reduce the drug effectiveness. In fact, a bypass mechanism explains why *Leishmania* parasites have low susceptibility to methotrexate or trimethoprim^21–23^, despite the fact that DHFR-TS gene knockout is lethal to the *Leishmania* parasites^24^.

Nare, Luba, and Beverly^22^ reported that upon dihydrofolate reductase-thymidylate synthase (DHFR-TS) inhibition, pteridine reductase 1 (PTR1) provides enough folate to guarantee the parasite survival. This finding suggested that both enzymes (DHFR-TS and PTR1) should be targeted to develop useful leishmanicidal drugs^17^. This hypothesis is still a matter of debate once the *ptr1* gene knockout proved lethal to the parasite, probably because reduced pterin, which is produced only by PTR1, is essential for parasite growth and metacyclogenesis^22^. Accordingly, some authors consider PTR1 a validated target for drug development and have pursued its inhibition^15^^,^^24^^,^^25^. Despite the advances in the field, the risk of selecting resistant strains was not addressed in those papers.

Another caveat of previous work is the fact that most of the biological data reported so far was obtained for PTR1 from *Leishmania major* (*Lm*PTR1), which causes cutaneous leishmaniasis, not the visceral form, as *L. chagasi* (*syn. L. infantum*) does. Despite the high overall sequence, similarities between *Lm*PTR1 and *Lc*PTR1 (90% – Figure S1), the active site of *L. chagasi* PTR1 is expected to be more flexible, once this enzyme is 100% identical to *L. donovani* PTR1, whose X-ray structure shows a disordered substrate binding site^26^. As a consequence, *Lm*PTR1 inhibitors may not be as effective against *Lc*PTR1.

In order to shed light on this matter, we expressed recombinant DHFR-TS and PTR1 from *L. chagasi* in *Escherichia coli*, so that standardized and balanced kinetic assays could be carried out. Next, we investigated 2,4-diaminopyrimidine derivatives as potential dual inhibitors for both enzymes, since derivatives of this chemical class have already been described as dihydrofolate reductase from *Schistosoma mansoni (Sm*DHFR)^27^^,^ *Lm*DHFR-TS and *Lm*PTR1^24^ inhibitors.

## Materials and methods

### Synthesis of 2,4-diaminopyrimidines

**Figure 1.**
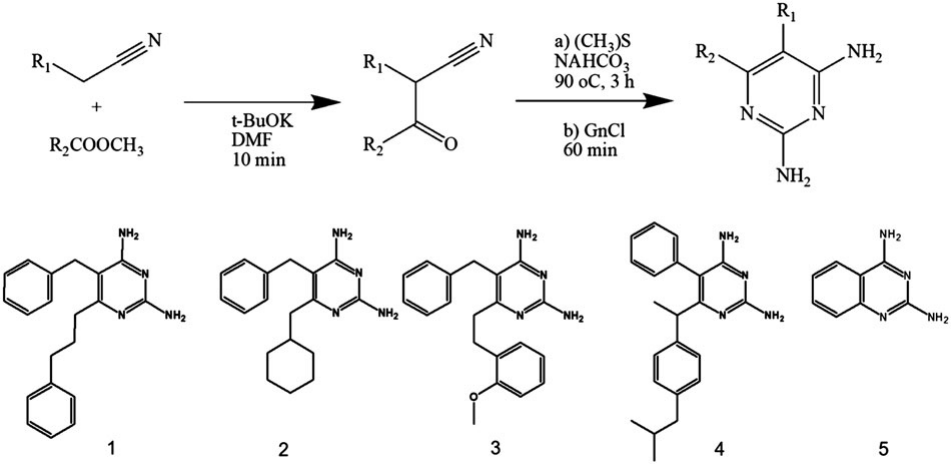
Major route steps to obtain 2,4-diaminopyrimidine derivatives **1**–**5.** Chemical structures are shown in the lower panel.

The 2,4-diaminopyrimidine derivatives were synthesized as described by Teles et al.^27^. Briefly, all compounds were obtained in moderate yields by the condensation of suitable ketonitriles and guanidine as described in Figure 1.

### Cloning of the enzyme LcPTR1

*Lc*PTR1 cloning was carried out through the ligation independent cloning (LIC) protocol (https://www.helmholtz-muenchen.de/fileadmin/PEPF/Protocols/LIC-cloning.pdf). The genomic sequence that codes for PTR1 from *L. chagasi*, available in Genbank server (Accession code XM_001465671.1), was employed to primers design. The Phusion system (New England Biolabs^®^) was used to amplify the DNA fragment containing the *Lc*PTR1 sequence using the forward and reverse primers 5′-**CAGGGCGCC**ATGGCTGCTCCGACCGTG-3′ and 5′-**GACCCGACGCGGT**TATCAGGCCCGGGTAAGGCT-3’, respectively, which were acquired from Exxtend (https://www.exxtend.com.br/site/). The nucleotide sequence (bold) required for the LIC cloning system use, was inserted in the sequence of the forward and reverse primers. The remaining primer sequences hybridize to *Lc*PTR1 gene. Genomic DNA from *Leishmania chagasi* cells (MHOM/BR/00/BA262), provided by Dr. Fábio Rocha Formiga (FIOCRUZ - Bahia) was extracted, as suggested in the UltraClean Tissue & Cells DNA Isolation kit protocol (Mobio, Carlsbad, CA, USA), and quantified at 260 nm wavelength (Nanodrop 200, Thermo Scientific, Waltham, MA, USA).

The polymerase chain reaction for *Lc*PTR1 gene amplification was carried out in the ProFlex 3x32-well thermal cycler (Applied Biosystems, Waltham, MA, USA). The following steps and conditions were employed: initial step at 95 °C for 3 min, followed by 35 cycles of denaturation step at 95 °C for 30 s, annealing at 43 °C for 30 s and a final extension step at 72 °C for 110 s. The PCR product was loaded on a 1% agarose gel for electrophoretic separation (100 volts for 70 min) and then purified using the kit Wizard^®^ SV Gel and PCR Clean-Up System (Promega, Madison, WI, USA). The purified product was quantified spectrophotometrically (Nanodrop 200, Thermo Scientific) at 260 nm wavelength.

The pETM11-LIC vector previously linearized by *BsaI* (NEB) digestion (60 min at 50 °C) and the insert were treated with T4 DNA polymerase for cohesive ends formation and then incubated (1:3) for 30 min at 25 °C for annealing. Then, *E. coli* (DH10β) chemically competent cells were transformed by heat shock^28^ and the product of the transformation was grown in Luria Bertani (LB) medium (tryptone 1%, sodium chloride 1%, yeast extract 0.5% and bacteriological agar 1.5%), supplemented with 30 μg/mL kanamycin, for 16 h at 37 °C.

True-positive clones were confirmed by colony PCR as follows: a colony-forming unit (CFU) was suspended in 100 μl type I sterile-water, which as then boiled for 5 min. The DNA from this suspension was employed as template material for PCR-amplification. The genetic material was PCR amplified using 1X PCR buffer (Invitrogen^®^, Madison, WI, USA), MgCl_2_ 1.5 mM, dNTP 0.2 mM, 0.2 μL Platinum^®^ Taq DNA polymerase (Thermo Scientific) and water (q.s. to 20 μL) and the T7 primers (forward: 5′-TAATACGACTCACTATAGGG-3′ and reverse: 5′-TAGTTATTGCTCAGCGGTGG-3′) and thermocycling parameters previously described for *Lc*PTR1 amplification.

The products of this reaction were analyzed on 1% agarose gel (100 V/1h) to confirm the presence of the gene’s amplification band (0.9 kbp) as true-positive construction. Positive clones were cultured for 12 h in LB broth, supplemented with kanamycin (30 μg/ml), at 37 °C and 150 RPM and then had their expression-plasmid DNA extracted with the aid of Pureyield Plasmid Miniprep System kit (Promega – A1223). The extracted plasmid was used for heat shock transformation^28^ of the chemically competent *E. coli* BL21 (DE3) cells. The expression-plasmid containing N-terminal His-tag, TEV cleavage site, and *Lc*PTR1 gene was sequenced using T7 primers and ABI 3500 Platform (Applied Biosystem) at Molecular Biology Research Laboratory (Pharmacy college – UFBA, Salvador, Brazil).

### Expression and purification of LcPTR1

A CFU previously grown on LB agar was inoculated into 3 ml of sterile LB broth supplemented with kanamycin (30 µg/ml) and kept under constant stirring (250 RPM at 37 °C for 12 h). After this time, the cell suspension was diluted (1: 100) in sterile LB broth (10 mg tryptone, 5 mg yeast extract and 5 mg NaCl per ml, pH 7) supplemented with antibiotics and kept under constant shaking (180 RPM) at 37 °C until OD_600nm_ = 0.6, at this point, the temperature was lowered to 25 °C, the culture was kept at constant shaking (180 RPM) for 18 h and multiple combinations of IPTG concentrations (0.05–1 mM) were evaluated for the soluble expression of *Lc*PTR1.

The cells were then recovered by centrifugation (16,000 X*g* at 4 °C for 15 min in a Sigma 1–14 K centrifuge) and resuspended in 50 mM Tris-HCl buffer pH 8.0 containing 125 mM NaCl and 20 mM imidazole supplemented by 1 mM phenylmethanesulphonylfluoride (PMSF P7626 Sigma Aldrich, Chicago, IL EUA) to mechanical lysis by sonication (15 cycles of sonication at 8 Watts for 15 s each, with 30 s intervals), in an ice-cold bath. The soluble fraction from lysate was recovered by centrifugation (14.500 X*g* at 4 °C for 15 min in a Sigma 1–14 K centrifuge). Next, the soluble fraction was filtered (0.45 μm – Sartorius) and loaded on crude His Trap FF column (GE Healthcare Life Sciences^®^, Chicago, IL EUA) previously equilibrated with 10 column volumes (CV) of 50 mM Tris-HCl buffer pH 8.0 containing 200 mM NaCl (buffer A). After the system was washed with 10 CV of buffer A supplemented with 20 mM imidazole, followed by 5 CV of buffer A supplemented with 50 mM imidazole. Finally, the *Lc*PTR1 was eluted with 5 CV of buffer A containing 500 mM imidazole.

All steps of the purification were monitored by 12% polyacrylamide gel electrophoresis (SDS-PAGE). The fractions containing the protein were pooled and the imidazole was removed after successive dilution steps (A 1:10 buffer) and centrifugation at with Amicon 30 kDa (Millipore, Chicago, IL EUA) a 4 °C e 3500 X*g*. Then, imidazole-free *Lc*PTR1 was incubated with *Tobacco Etch Virus* protease (TEV), 1:20 (M/M) ratio, at 4 °C for 12 h to cleavage of N-terminus His tag. The proteolysis product was loaded on a Nickel-sepharose column (GE Healthcare^®^), previously equilibrated with buffer A (10 CV).

The cleaved *Lc*PTR1, collected in the void, had its concentration measured using Bradford reagent^29^ (595 nm) (Figure S2). All purification steps were monitored by 12% SDS-PAGE.

### Cloning of LcDHFR coding gene into E. coli ArticExpress (DE3) cells

The coding sequence for *Leishmania chagasi* strain JPCM5 DHFR-TS available in Genbank server (Accession code: XM_001463132.2) plus 23 nucleotides flanking the gene was employed for primer design and cloning into pET28a vector.

Restriction sites were assessed using the NebCutter server (http://nc2.neb.com/NEBcutter2/). Genomic DNA from *Leishmania chagasi* (MHOM/BR/00/BA262) promastigotes was extracted, as suggested in the UltraClean Tissue & Cells DNA Isolation kit protocol (Mobio) and quantified using *Qubit* fluorimeter and *Qubit dsDNA BR* kit (Invitrogen).

The *Lc*DHFR-TS coding region was PCR-amplified using the genomic DNA, as a template, and the primers described next: The forward primer includes the NdeI restriction site (underlined) 5′-CAATACGCATATGTCCAGGGCAGCTG-3′ and the reverse primer includes the *Hind*III restriction site (underlined) 5′-GCCTCCAAGCTTTCTTAACGGCCATC-3′. The restriction site for the NdeI enzyme was positioned in the 5' region of the *Lc*DHFR-TS gene, which allowed the expression of the enzyme containing the polyhistidine tail in the N-terminal domain of the protein.

The PCR mixture included 70 ng (1 µL) of genomic DNA, 1× HF buffer (Thermo Scientific^®^) buffer, 1.5 mM MgCl_2_, 0.2 mM dNTP, 0.5 μM each primer and water q.s. to 50 μL. The gene amplification reaction was carried out in the ProFlex 3x32-well thermal cycler (Applied Biosystems) using the following parameters: single denaturation step (94 °C for 2 min), followed by 35 cycles of denaturation at 94° C for 30 s, annealing at 56.2 °C for 30 s and extension at 72° C for 110 s. PCR included a final extension at 72 °C for 600 s.

The reaction product was loaded on a 1% agarose gel for electrophoretic separation (100 Volts for 70 min). Next, the amplicon was purified directly from the gel matrix with Gene Jet Gel extract and DNA cleanup kit (Thermo Scientific^®^) and quantified spectrophotometrically (Nanodrop 200, Thermo Scientific) at 260 nm wavelength.

Next, the purified DNA fragment and pET28a vector were individually double-digested with *Nde*I and *Hind*III FastDigest restriction enzymes (Thermo Scientific) and then ligated (5:1 insert/vector ratio) in the presence of T4 DNA ligase (Thermo Scientific) according to manufacturer instructions. *E. coli* ArticExpress (DE3) cells were transformed with the recombinant plasmid by heat shock protocol^28^. Colonies containing the inserted gene were selected using the resistance markers to kanamycin and gentamycin. True-positive clones were confirmed by colony PCR (using T7 primers: forward: 5′- TAATACGACTCACTATAGGG- 3′ and reverse: 5′-TAGTTATTGCTCAGCGGTGG-3′as described previously. The amplification parameters were set as follows: single initial denaturation step at 94 °C for 180 s followed by 35 cycles of denaturation at 94 °C for 45 s, annealing at 42.5 °C for 30 s and extension at 72 °C for 110 s. A final extension step (72 °C for 600 s) was carried out complete the reaction. The amplification products were analyzed by 1% agarose gel electrophoresis and those 1718 bp-compatible amplified DNAs (1569 bp from insert plus 149 bp from the vector) was submitted to DNA sequencing using T7 primers on ABI 3730 Platform (Applied Biosystem) at Myleus Biotechnology center (Minas Gerais/Brazil). Colonies transformed with mutation-free plasmid were employed for the following steps.

### Expression and purification of LcDHFR-TS

A CFU, previously grown on LB agar supplemented with kanamycin (30 µg/mL) and gentamycin (20 µg/mL) was cultivated overnight in antibiotic-supplemented LB broth (peptone from gelatin 1%, yeast extract 0,5%, sodium chloride 1% adjusted to pH 7,5), at 37 °C and 200 RPM. After that, the cellular suspension (pre-inoculum) was employed to inoculate a freshly prepared sterile antibiotic-supplemented LB broth (1:100), which was kept at 37 °C and 200 RPM until OD_600nm_ = 0.6. At this point, the temperature was lowered to 10° C and IPTG was added (0.5 mM). After 48 h, the cells were then recovered by centrifugation (4000 X*g* at 4 °C for 10 min) and resuspended in 50 mM phosphate buffer pH 7.0, containing 200 mM (NH_4_)_2_SO_4_ supplemented with protease cocktail inhibitors 0,1X (SigmaFAST^®^ - S8830), for mechanical lysis by sonication (15 cycles of sonication at 8 Watts for 30 s each, with 30 s intervals, using Sonics Vibra-Cell Model VCX130PBt), in an ice bath.

Next, cellular debris and insoluble proteins (sediment) were separated from the supernatant by centrifugation (14.500 X*g* at 4 °C for 30 min in a Sigma 1–14 K centrifuge) and the soluble fraction was loaded on Ni sepharose His-trap fast flow resin (GE Healthcare) previously equilibrated with 5 CV of buffer A (50 mM phosphate buffer pH 7.0 containing 200 mM (NH_4_)_2_SO_4_ and 50 mM imidazole). 200 mM ammonium sulfate was added to the purification protocol and stock solution to increase the stability of the protein^30^.

After contaminants were eluted with 30 CV of buffer A, *Lc*DHFR-TS was eluted by linear increase of imidazole concentration (50–500 mM) within buffer A. The fractions containing the protein were pooled together and the imidazole was removed by successive dilution and centrifugation steps (3000 X*g* at 4 °C for 30 min) in 50 mM phosphate buffer pH 7.0 containing 200 mM (NH_4_)_2_SO_4_. Imidazole-free *Lc*DHFR-TS had the N-terminus polyhistidine tag removed by thrombin (*Sigma* Cat. T7513) digestion at 25 °C for 2.5 h (0.1:5 Thrombin Unit: *Lc*DHFR-TS mg). Cleaved protein was recovered by loading the digested protein solution onto Ni sepharose His-trap resin (GE Healthcare), previously equilibrated with 50 mM phosphate buffer pH 7.0 containing 200 mM of (NH_4_)_2_SO_4,_ and eluting it with 20 ml of the same buffer. All purification steps were monitored by 12% SDS-PAGE. The *Lc*DHFR-TS had its concentration measured using Bradford reagent^29^ (595 nm) (Figure S2). The amount of contaminant thrombin present in the final solution resulting from the *Lc*DHFR-TS purification protocol is approximately 0.5% in weight (0.0062 mg of thrombin per *Lc*DHFR-TS mg). Aliquots of the purified enzyme were stored at -80 °C in the presence of 30% glycerol (*v/v*) and thawed immediately before use.

### Kinetic studies with LcDHFR-TS and LcPTR1

#### LcDHFR-TS apparent Km determination

Kinetic measurements were carried out using a standard assay^31^, at pH 7.0 (50 mM sodium phosphate). Briefly, NADPH consumption was monitored spectrophotometrically (340 nM) (Shimadzu^®^ UV-1800, Columbia MD, USA) at constant temperature (25 °C) and the absorbance variation within the first 30 s was employed to calculate the initial rate of reaction, by linear regression of the raw data.

The appropriate concentration of enzyme to be employed in the kinetic assays was evaluated by observing the reaction time required for substrate depletion under enzyme concentrations of 1.0 and 2.0 μM in the presence of 50 μM of DHF and NADPH.

The effect of the pH (5.0–8.0) over enzymatic activity was investigated with 0.2 μM of *Lc*DHFR-TS and saturating NADPH and DHF concentrations (100 μM) (50 mM citrate buffer was used for pH 5.0 activity assay. 50 mM sodium phosphate buffer was used for pH range 6.0 to 8.0 activity assay). After the optimal pH was identified the apparent Km of the cofactor was determined by following a decrease in absorbance (340 nm) at growing up the concentration of NADPH (0.39 to 50 μM) at saturating concentration of DHF (100 μM). Likewise, substrate apparent Km was determined at saturating NADPH concentration (50 μM) and varying DHF concentrations (0.20–12 μM). All the measurements were carried out in triplicate and the apparent Km values for the cofactor and substrate were determined under a pseudo first-order kinetics assumption using least-squares non-linear regression method, as available in the GraphPad Prism version 5.0 for Windows (GraphPad^®^ Software, San Diego, CA, USA, www.graphpad.com).

#### LcPTR1 apparent Km determination

Kinetic measurements were performed according to the protocol described^32^ via spectrophotometer (Shimadzu UV-1800) at pH 4.7 (20 mM sodium acetate). NADPH consumption was monitored spectrophotometrically (340 nm) at constant temperature (30 °C). The variation of the absorbance monitored for 60 s was used to calculate the initial rate of the reaction by linear regression.

The apparent Km of NADPH was determined by varying its concentration (6–300 μM), maintaining the biopterin at saturation concentration (100 μM). Likewise, the apparent Km of the substrate was determined by saturation of NADPH concentration (100 μM) and increasing concentrations of biopterin (3.12–100 μM).

All measurements were performed in triplicate and the apparent values of Km for the cofactor and substrate were determined using the non-linear regression method (3-parameter equation), as available in GraphPad Prism version 5.0 for Windows (GraphPad^®^ Software, San Diego, CA, USA, www.graphpad.com).

#### Single concentration inhibition assays

The *in vitro* screening of putative inhibitors was carried out in balanced conditions: *Lc*PTR1 0.4 μM, biopterin 25 μM, NADPH 35 μM in 20 mM sodium acetate buffer (pH 4.7); *Lc*DHFR-TS: 0.2 μM, DHF 5.0 μM, NADPH 5.0 μM in 50 mM sodium phosphate buffer (pH 7.0).

Comparison of the enzymatic activities in the absence and presence of the compounds (50 μM), which were dissolved in DMSO (5% *v/v* final concentration), was employed to calculate the percent inhibition (Equation (1))

(1) $$\%\text{ Inhibition}=\text{1}00\;-\;\left({\text{V}}_{\text{i}}/{\text{V}}_{\text{c}}\right)\times \text{1}00$$

where V_i_ is the initial velocity in the presence of the inhibitor and V_c_ is the reaction rate in the presence of DMSO (5% (*v/v*)).

#### Determination of IC_50_ values

The compounds that inhibit either L*c*PTR1 or *Lc*DHFR activity ≥30%, in the single concentration assays, had their IC_50_ values determined by making rate measurements for at least five inhibitor concentrations.

Initial velocity measurements (V_i_) were employed to calculate inhibition percentages, whose plot versus log of inhibitor concentration afforded the IC_50_ values, by nonlinear regression (assuming Hill constant = 1.0) as available in the GraphPad program Prism version 5.0 (GraphPad^®^ Software, San Diego, CA, USA, www.graphpad.com).

#### Determination of the inhibition mechanism

The type of inhibition was determined under the same reaction conditions described above for different inhibitor concentrations (0.0–20.0 μM) at five varying substrate concentrations (biopterin: 6.25, 12.5, 25, 50 and 100 μM; DHF 0.78, 1.56, 3.12, 6.25, 12.5 μM), whereas the cofactor concentration was at saturating conditions (*Lc*PTR-NADPH = 100 μM; *Lc*DHFR-TS-NADPH  = 50 μM).

Next, the kinetic measurements were carried out in saturating substrate concentrations (biopterin: 100 μM – DHF: 12.5 μM) and varying the concentration of NADPH (6.25, 12.5, 25, 50 and 100 μM). Apparent Km values, calculated by non-linear regression, were compared by *T*-test, available in GraphPad Prism version 5.0, and considered significantly different for *p*<.05. The results of this analysis were plotted in double-reciprocal graphs for visual analysis purposes only. The dissociation constant (*K_i_*) of the active derivatives against both enzymes was obtained through Cheng–Prussov equation for competitive inhibitors^33^, as follows:

(2) $${\mathrm{IC}}_{50}=K_{i}\left(1+\frac{\left[S\right]}{K_{m}}\right)$$

where IC_50_ is the inhibitor concentration required to reduce enzyme activity by 50%, [*S*] is the substrate concentration used in the inhibition assay and Km is the apparent Michaelis constant for the substrate in the absence of the inhibitor, at saturating NADPH concentration.

## Results and discussion

### Cloning, expression, and purification of LcPTR1

The complete coding sequence for *Lc*PTR1 from *L. chagasi* plus eight nucleotides flaking the gene was employed for primer design, which began with the identification of restriction enzymes that would not cleave the *Lc*PTR1 gene.

**Figure 2.**
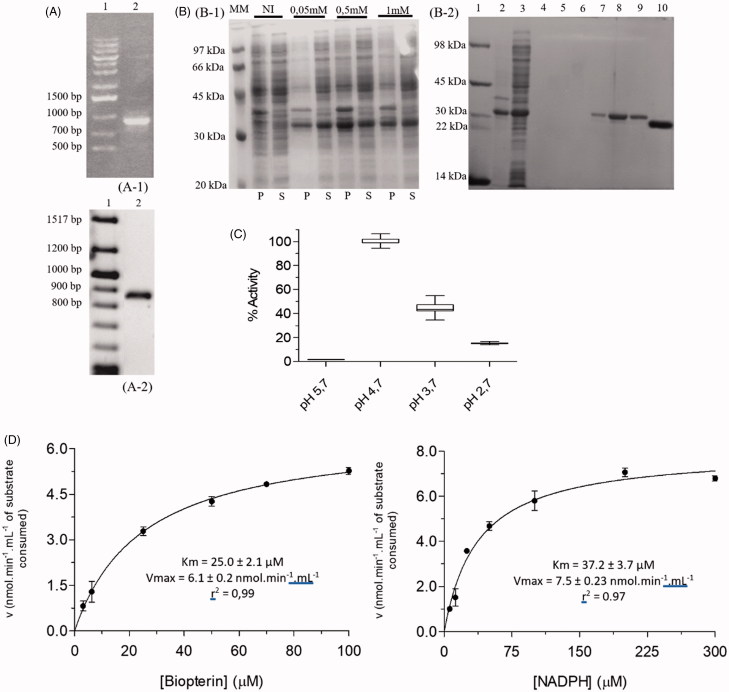
Cloning, expression, purification, and kinetic characterization results for *Lc*PTR1. (A) Cloning steps to insert *Lc*PTR1 gene into pETM11 expression vector monitored by 1% agarose gel. A1: (1) Marker 1 kb DNA Ladder, A1: (2) PCR amplification product with base pairs (bp) equivalent to *Lc*PTR1 gene; A2: (1) Tridye 100pb ladder (NEB); A2: (2) Product of PCR reaction employing *E. coli* CFU resulting from the transformation protocol. (B) Expression and purification of *Lc*PTR1. SDS/page 12%. (B1) Protein expression profile of *Lc*PTR1 in *E. coli* BL21 (DE3). After expression induction at 25 °C (18 h), in the presence of different IPTG concentrations (0.05, 0.5, and 1.0 mM). NI: the absence of IPTG; P: pellet: S: supernatant; MM: LMW-SDS GE molecular weight standard (kDa). B2: (1) molecular weight standard (kDa); (2) purification supernatant; (3) fraction 20 mM imidazole; (4–6) 50 mM imidazole; (7–9) 500 mM imidazole. (10) *Lc*PTR1 after cleavage. (C) Activity of *Lc*PTR1 in different pH ranges. Values represent median and interquartile range of % activity when compared to the highest median of activity obtained, at pH 4, 7 (*N* = 3). (D) Apparent Km determination for recombinant *Lc*PTR1.

The amplification reaction afforded a single product of 867 bp (Figure 2(A)), which, following purification, was cloned into the pET-M11 vector using the LIC system.

This strategy allows the expression *Lc*PTR1 fused to a N-terminal His-tag. A similar approach was employed to obtain the PTR1 from *Leishmania donovani* in the soluble form^34^. Following the transformation of competent *E. coli* BL21 (DE3) cells and “colony PCR” screening, a positive and sequence verified (Figure S3) colony was selected for protein expression studies.

After expression of *Lc*PTR1 was carried out at 25 °C, 18 h, 0.5 mM IPTG, a single-step purification, using affinity chromatography, that affords heterologous protein with high purity (Figure 2(B)), once this tag can influence protein stability and/or catalytic properties^35^.

TEV protease was employed to remove it and allow *Lc*PTR1 to be obtained, after another round of affinity chromatography. This protocol affords 5-fold more protein (15 mg.L^−1^ of bacterial culture) than the one described for *Ld*PTR1^34^ and a similar yield as the one described for *Lm*PTR1^36^.

### Cloning, expression and purification of LcDHFR-TS

**Figure 3.**
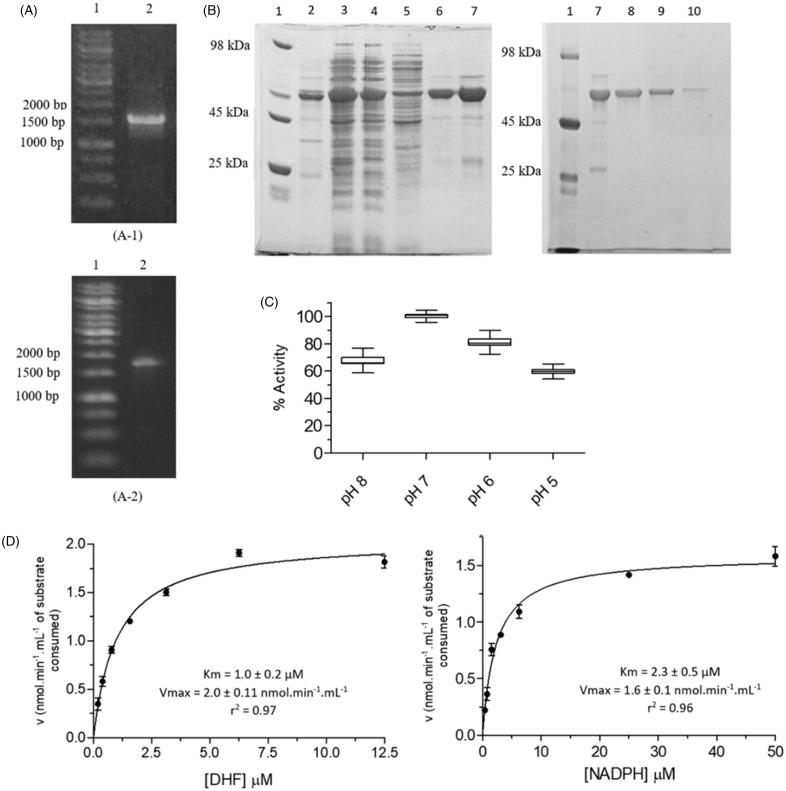
Cloning, expression, purification and kinetic characterization results for *Lc*DHFR-TS. (A) Cloning steps of the *Lc*DHFR-TS gene in the pET28a vector, monitored by 1% agarose gel. A1, A2: (1) Marker 1 kb DNA Ladder; A1: (2) Product of the amplification reaction of the *Lc*DHFR-TS gene from the genomic DNA; A2: (2) Product of PCR reaction with primer T7 employing CFU from the transformation protocol. (B) Expression and purification of *Lc*DHFR-TS. Gel SDS-page 12%. (1) Molecular weight standard; (2–3) insoluble and soluble fraction of the lysate; (4–5) contaminants eluted in low imidazole concentration; (6–7) Wash with buffer containing 500 mM imidazole; (8–10) Column eluate with imidazole-free buffer after application of the dialyzed material and incubated with thrombin. (C) Activity of *Lc*DHFR-TS in different pH ranges. Values represent median and interquartile range of % activity when compared to the highest median of activity obtained, at pH 7.0 (*N* = 3). (D) Apparent Km determination for *Lc*DHFR-TS.

The migration profile of the PCR product shows a band that is consistent with the expected amplicon containing the *Lc*DHFR-TS gene (1563 bp), plus the extension-primers (23 bp) (Figure 3(A)). After purification of the PCR product, the amplified insert (1.2 μg (46 ng/μl)) was cloned into the pET28a expression vector using the restriction site for the *Nde*I enzyme positioned in the 5' region of the *Lc*DHFR-TS gene.

This strategy allows the expression of the recombinant protein fused to His-tag in the N-terminal domain to facilitate its purification through nickel resin affinity chromatography. The same N-terminal His-tag fusion strategy was successfully employed in the cloning, expression, and purification of *Leishmania* (*Vianna*) *braziliensis*^37^, and *T. cruzi* DHFR-TS^38^.

Finally, heat shock transformation protocol allowed to isolate sequence verified (Figure S4) colony-forming units containing the gene that codes for *Lc*DHFR-TS (Figure 3(A)). Initial *Lc*DHFR-TS expression tests using *E. coli* BL21 (DE3) showed that the recombinant protein was observed predominantly in the insoluble fraction of the cell lysate (Figure S5). Low expression profile was reported for *L. major* DHFR-TS when BL21 strain was employed ^39^. In order to increase the yield of a soluble and correctly folded recombinant protein that might be obtained, the Arctic Express *E. coli* (DE3) strain was employed^40^. This strategy proved successful and allowed us to obtain soluble *Lc*DHFR-TS when the cells were grown at 10 °C, for 48 h, in the presence of IPTG 0.5 mM (Figure 3(B), lanes 2 and 3). Several DHFT-TS are susceptible to protease action (*T. brucei* DHFR-TS^41^^,^^42^, *L. tropica* DHFR-TS^31^, and *L. major* DHFR-TS^39^).

Likewise, proteolytic cleavage of *Lc*DHFR-TS was also observed in the present work (Figure 3(B), lane 7), even upon addition of protease inhibitors cocktail (SigmaFAST) to the cell lysis buffer. Nevertheless, chromatographic purification by affinity made it possible to obtain the partially purified protein (Figure 3(B), lanes 6–7), which has its His-tag cleaved with thrombin. Non-cleaved *Lc*DHFR-TS was removed by a second elution on nickel sepharose resin (Figure 3(B), lanes 8–10), as described for *Lc*PTR1. Hence, the overall yield of *Lc*DHFR-TS is 10.0 mg.L ^−1^ of bacterial culture.

### Kinetic characterization of LcDHFR-TS and LcPTR1

#### Catalytic activity of LcDHFR-TS

Absorbance decrease at 340 nm over time (350 s) after the start of the reaction showed a linear profile up to 300 s when used 1.0 μM of the enzyme, indicating the steady-state condition is reached in the assays for at least the initial 30 s of the assay.

In addition, in order to determine optimum assays conditions for *Lc*DHFR-TS kinetic characterization and inhibition, it was necessary to evaluate the effect of pH on the catalytic activity, since medium pH affects protonation states of amino acids related to catalysis and/or intermolecular interactions^43^.

The results obtained showed that catalytic activity of the DHFR domain of *Lc*DHFR-TS is influenced by pH in the range of 5–8. Among the pH ranges evaluated, the maximum catalytic activity was observed at pH 7.0 (Figure 3(C)), that explains why the next steps were carried out on this pH.

Intrategumental pH studies of *L. donovani* observed that the parasite maintains neutral intracellular pH^44^, even under acidity or basicity medium conditions, thus demonstrating that the assay has biological significance as it approaches the enzyme natural habitat. Studies with DHFR-TS from different organisms agree with this data when describing the optimum pH at 7.0 for *L. major*^45^ and *Plasmodium vivax*^46^ enzymes. Other studies indicate that the optimum pH for the DHFR-TS of *Trypanosoma brucei*^41^ and *Plasmodium berghei*^47^ is in the pH range of 6.5–8.0. On the other hand, some studies show that for vertebrates and certain bacteria, catalytic activity is promoted at lower pH values^48^.

Although the decrease in the pH of the medium tends to promote catalytic activity by facilitating the transfer of the hydride to the nitrogen 5 of the DHF pteridine ring and consequently, increase Vmax^49^, this is not observed for DHFR-TS cases mentioned above, probably due to structural factors.

Once reaction conditions were standardized, we proceeded to *Lc*DHFR-TS kinetic characterization. Determination of kinetic parameters is crucial to the standardization of assay conditions for inhibitors screening^50^ since cofactor and substrate concentrations must be close to the Km apparent in the assay to allow identification of different inhibitors modalities: competitive, noncompetitive, and uncompetitive^43^. As an ordered bi-random mechanism is described to the formation of DHFR • NADPH • DHF complex^51^, the simplifications described by Michaelian kinetics can be applied for the *Lc*DHFR-TS by maintaining saturating conditions of one substrate to allow kinetic characterization of the other^33^.

The values obtained for Km^app^ and Vmax^app^ for DHF were 1.0 ± 0.2 μM and 2.0 ± 0.11 nmol.min^−1^.mL^−1^, respectively, whereas for NADPH, Km and Vmax values were 2.3 ± 0.5 μM and 1.6 ± 0.1 nmol.min^−1^.mL^−1^, respectively (Figure 3(D)). The Km^app^ values of *Lc*DHFR-TS are close to the described for DHFR-TS of other species of *Leishmania* genus such as *L. tropica*, with 1.5 μM and 2.7 μM^39^, and *L. major*, 1.6 μM and 0.45 μM^31^ for DHF and NADPH, respectively, what are already expected since the differences in its catalytic regions of the sequences are punctual. Both data obtained here for *L. chagasi* and those reported for *L. tropica* DHFR-TS shows lower affinity values of cofactor relative to the substrate for the enzyme, such a feature can be exploited by designing inhibitors directed to bind in the cofactor site, competing with NADPH, since its lower affinity would imply in lower concentration of the inhibitor needed to displace it from the enzyme site.

When we consider the number of catalytic turnover events (k_cat_)^52^, *Lc*DHFR-TS presents a k_cat_ of 0.033 s^−1^. Among DHFR-TS from other organisms, *L. major*^31^ and *T. brucei*^42^ k_cat_ constants, 27 s^−1^ and 26 s^−1^, respectively, are higher than that of the *L. chagasi* enzyme (0.033 s^−1^).

#### Catalytic activity of LcPTR1

The enzymatic reaction monitored through the decrease in absorbance (340 nm) showed a linear profile for up to 600 s when using 0.4 μM of *Lc*PTR1 which indicates steady-state conditions in the assays for at least 60 initial seconds of the assay.

Next, in order to determine optimum assays conditions for *Lc*PTR1 activity, its dependence on pH variation was examined. Significant variation of *Lc*PTR1 activity was observed in the pH range between 2.7 and 5.7 (Figure 2(C)). As a marked peak of activity was observed at pH 4.7, this was then considered as the optimum pH for *Lc*PTR1 catalytic activity. Similarly, the pH of 4.7 was determined as optimal for *Lm*PTR1^22^ and for *Ld*PTR1, the optimum pH is 4.8^53^.

In addition to the reaction medium pH, the substrate and cofactor concentrations are critical for the establishment of balanced assay conditions in kinetic assays employed for potency and mechanism of inhibition determinations. Such conditions are achieved when the kinetic reaction medium presents substrate and cofactor concentrations close to the Km, since these concentrations can influence the identification of competitive, non-competitive, or uncompetitive inhibitors^52^.

Accordingly, as PTR1 presents an ordered mechanism of PTR1•NADPH•BPT complex formation^23^, in which the substrate (BPT) only binds the enzyme after formation of PTR1•NADPH, it is possible for the use of the Michaelian simplifications for the kinetic characterization of *Lc*PTR1 by maintaining saturating conditions for BPT to enable the determination of cofactor Km, and vice versa^33^.

The values obtained from Km^app^ and Vmax^app^ for BPT were 25 ± 1.2 μM and 13.0 ± 0.4 nmol.min^-1^.mL^-1^, respectively, whereas for NADPH, the values of Km and Vmax were 37.48 ± 2.1 μM and 15.4 ± 0.6 nmol.min^-1^.mL^-1^ (Figure 2(D)), respectively. The following values were reported for *Ld*PTR1: BPT Km^app^=5.8 μM; NADPH Km^app^=18.5 μM^54^. It is worth mentioning that these values were obtained through the double-reciprocal plot, which according to Copeland^33^ can infer errors of up to 20%, in addition, the protein used to determine the kinetic constants were fused with His-tag.

The kinetic parameters found for *Lc*PTR1 substrate and cofactor are not similar to the values reported for other species of *Leishmania*: for *L. tropica* PTR1^55^, was found for BPT Km  = 3.5 µM and for NADPH Km  = 19.0 µM, while *L. major* PTR1^22^ were found Km of 4.6 and 6.7 μM for BPT and NADPH, respectively.

However, the values found for *Lc*PTR1 agree with the crystallographic data of *Ld*PTR1, since a disordered active site must have lower affinity for its ligands^26^. Such PTR1 kinetic data show a lower affinity for the cofactor relative to the substrate, this pattern is also observed for *L. tarantoleae* PTR1 (BPT/NADPH Km^app^=3.5/19)^55^ and *L. major* (BPT/NADPH Km^app^=4.6/6.7) ^56^.

Comparing *Lc*PTR1 and *Lc*DHFR kinetic characterization data, both enzymes show lower affinity for the cofactor relative to the substrate, which is evidenced by higher Km^app^ values for NADPH (*Lc*DHFR: DHF/NADPH Km^app^=1.0/2.3; for *Lc*PTR1: BPT/NADPH Km^app^=25.0/37.5). When we consider the number of catalytic turnover events per time (k_cat_)^50^, the *Lc*PTR1 presents a k_cat_ of 0.54 s^-1^. Among PTR1 from other organisms, *L. major* PTR1^56^ k_cat_ is similar to the *L. chagasi* enzyme (0.44 versus 0.54 s^−1^, respectively), whereas the for *T. brucei* PTR1^32^ k_cat_ is higher than that of *L. chagasi* (4.3 versus 0.54 s^−1^, respectively).

Although the k_cat_ values are higher for *Lc*PTR1 with respect to *Lc*DHFR-TS (0.54 versus 0.033 s^−1^, respectively), when the catalytic efficiency is considered (k_cat_/km ratio), the *Lc*DHFR-TS presents higher constant value (3.3 × 10^4^ versus 2.1 × 10^4^M^−1^s^−1^, respectively). This pattern is also observed for *L. major* (PTR1 5.8× vs DHFR-TS 21 × 10^6^M^−1^s^−1^, respectively)^31^^,^^56^ and *T. brucei* (PTR1 4.3 × 10^5^ vs. DHFR-TS 6.8 × 10^6^, respectively)^32^^,^^42^. Hence, our data suggest that *Lc*DHFR-TS recognizes its substrate more efficiently than *Lc*PTR1.

#### LcPTR1 and LcDHFR inhibition assays

Although PTR1 and DHFR are not related at the primary sequence level, both enzymes are inhibited by compounds containing the 2,4-diaminopyrimidines skeleton^24^ which can be explained by the chemical similarity between the natural substrate of PTR1 and DHFR-TS and the 2,4-diaminopyrimidine nucleus.

**Figure 4.**
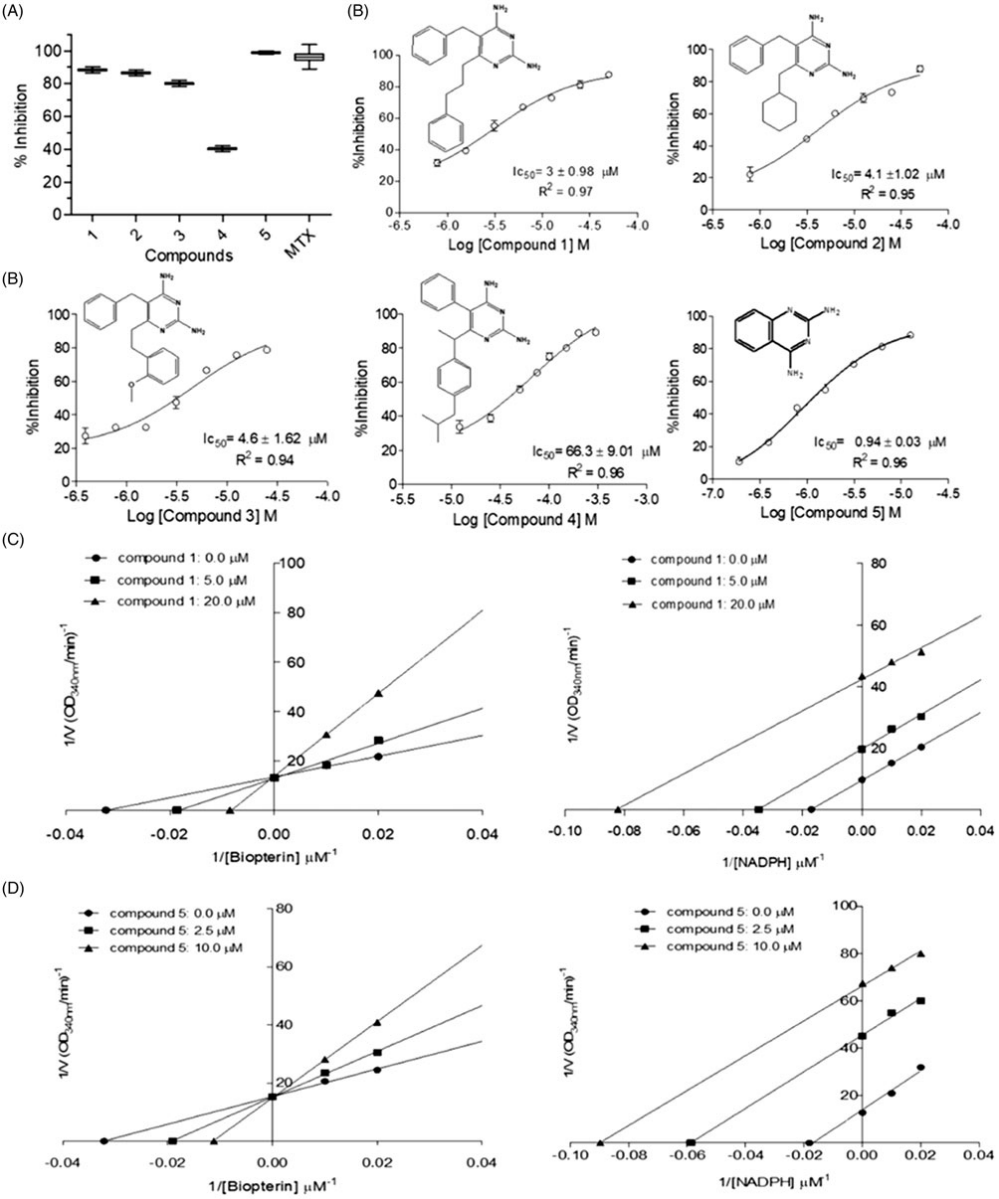
Kinetic inhibition assays results for *Lc*PTR1. (A) Single concentration (50 μM) inhibition assay. (B) Dose-response curves for compounds 1–5 against *Lc*PTR1. IC_50_ values calculated by non-linear regression in GraphPad Prism^®^ 5.0 software. (C) Effect of compound 1 over NADPH and BPT kinetic constants (Km^app^ and Vmax^app^). *Statistical difference of results were considered when *p* < .05 (Kruskal–Wallis ANOVA). (D) Effect of compound 5 over NADPH and BPT kinetic constants (Km^app^ and Vmax^app^) *Statistical difference of results were considered when *p* < .05 (Kruskal–Wallis ANOVA).

**Figure 5.**
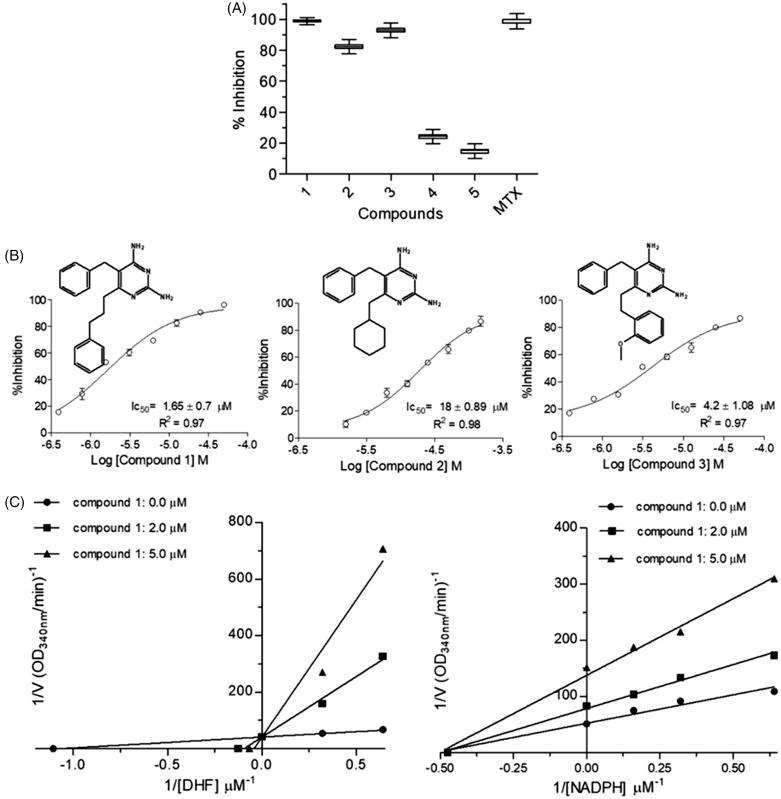
Kinetic inhibition assays results for *Lc*DHFR-TS. (A) Inhibition profile against in 50 μM inhibitor single concentration assay. (B) Dose-response curves for compounds 1–3 against LcDHFR-TS. IC_50_ values calculated by non-linear regression in GraphPad Prism^®^ 5.0 software. (C) Effect of compound 1 over NADPH and DHF kinetic constants (Km_app_ and Vmax_app_). *Statistical difference of results were considered when *p* < .05 (Kruskal–Wallis ANOVA).

Once the inhibition of both enzymes may have a synergistic detrimental effect over the parasite survival^17^, and prevent the selection of resistant strains, we decided to assay diaminopyrimidine derivatives, which have already been described as *S. mansoni* DHFR-TS inhibitors^27^, against *Lc*PTR1 and *Lc*DHFR-TS. Single-dose assays (Figure 4(A) and 5(A)) suggest that compounds **1** and **3** are active against both enzymes (e.g. compound **1** × *Lc*PTR1 = 90 ± 1% vs. *Lc*DHFR-Ts  = 100 ± 1.17%), whereas compounds **2** and **4** are more active against *Lc*PTR1 than *Lc*DHFR (*Lc*PTR1 inhibition  = 40 ± 1%, vs. *Lc*DHFR inhibition  = 20 ± 2.4%) and compound **5** seems to inhibit *Lc*PTR1 (98 ± 0.8% inhibition at 50 µM) but not *Lc*DHFR (20 ± 2.4% inhibition at 50 µM). However, this preliminary analysis might be misleading once the catalytic efficiency of *Lc*PTR1 and *Lc*DHFR-TS are significantly different. In this case, IC_50_ values are suitable only to compare the compounds´ potency for one specific target, either *Lc*PTR1 or *L*cDHFR-TS. For instance, compounds **1**, **2**, **3** show a discrete variation in IC_50_ values against *Lc*PTR1 (Figure 4(B)).

This result suggests that structural variations at position 6 of the 2,4-diaminopyrimidine nucleus (Figure 4(B)) appear not to influence the potency of these compounds against this target. On the other hand, the activity profile of compounds **1**–**3** against *Lc*DHFR-TS (Figure 5(B)) suggests that the presence of an aromatic ring at least two carbons away from the 2,4 diaminopyrimidine nucleus is crucial for *Lc*DHFR-TS inhibition (see IC_50_ of compounds **1** vs. **2**).

Although the IC_50_ values of compound **1** are lower for *Lc*DHFR-TS than *Lc*PTR1, one cannot say it is more potent against the first, since substrate concentration, reaction pH, temperature and Km of each enzyme can influence the value of IC_50_^57^,^58^. In order to overcome this limitation, *K*_i_ values calculated with the Cheng-Prussov equations might be employed, as long as the compounds´ mode of inhibition was known. Consequently, we undertake a careful investigation of compounds inhibition mode. Once compounds **1**–**4** possess high structural similarity, it is reasonable to assume that they share the same mechanism of inhibition. Thus, just results for compound **1** and **5** are discussed next.

### Mechanism of inhibition assays

Kinetic parameters like Km^app^ and Vmax^app^ under different inhibitor concentrations were analyzed in order to determine the mechanism of action of compound **1** over *Lc*PTR1. Results obtained with saturating concentration of NADPH (Figure 4(C)), show a set of lines that intercept the Y-axis at the same point (1/Vmax^app^) and cross the X-axis (-1/Kmax^app^) at different points.

This behavior suggests a competitive mechanism of inhibition, but the fact that PTR has a bi-bi ordered catalytic mechanism^23^ requires that compound **1** also shows an uncompetitive mechanism of inhibition to the cofactor.

In order to confirm this hypothesis, enzymatic inhibition experiments were carried out at a saturating concentration of biopterin, while the NADPH concentration was varied (Figure 4(C)). As expected, the values of Km^app^ and Vmax^app^ decrease proportionally, as the concentration of compound **1** increases. Thus, the graph of the reciprocal double shows parallel lines, typical of an inhibitor uncompetitive concerning the cofactor.

As the structure of compound **5** differs substantially from the other compounds, we decided to perform experiments to determine the mechanism of inhibition to this compound as described above.

Thus, when the assays were performed with saturating concentration of NADPH and different concentrations of biopterin, it was observed that the lines intersect the Y-axis at the same point, while the intersection with the X-axis occurs at different points (Figure 4 (D)). Therefore, compound **5** exhibits the same behavior observed for compound **1** (competitive mechanism with biopterin). In fact, when different concentrations of NADPH were used, reduced values of Km^app^ and Vmax^app^ were observed (Figure 4 (D)). These results confirm that compound **5** has an affinity for the PTR1-NADPH complex (mechanism of inhibition uncompetitive with the cofactor).

When the assay was carried out with *Lc*DHFR-TS, it was observed that DHF Km^app^ increases linearly as compound **1** concentration (Compound **1**=0 µM – Km  = 1.0 ± 0.2 µM; **1**=2 µM – Km  = 7.8 ± 1.2 µM; **1**=5 µM – Km  = 15.0 ± 4.8 µM), whereas its Vmax^app^ is not affected by increasing concentrations of compound **1** (Vmax  = 1.9 ± 0.1 nmol.min^−1^.mL^−1^).

On the other hand, NADPH Km^app^ remains the same (Km  = 2.1 ± 0.3 µM) but its Vmax^app^ decreases linearly when increasing concentrations of compound **1** are added to the catalytic reaction (Compound **1**=0 µM – Vmax  = 1.6 ± 0.1 nmol.min^−1^.mL^−1^; 2 µM – Vmax  =  1.0 ± 0.1 nmol.min^−1^.mL^−1^, 5 µM – Vmax  = 0.5 ± 0.05 nmol.min^−1^.mL^−1^) (Figure 5(C)).

The competitive mechanism related to substrate observed for compound **1** is consistent with the data described for 2,4-diaminopyrimidine inhibition of *L. major* enzyme^59^. The same pattern was also observed for *Bacillus anthracis*^60^ and *Schistosoma mansoni*^27^ enzymes. On the other hand, although the noncompetitive inhibition related to cofactor is also observed against *S. mansoni*^27^ and *Gallus gallus*^59^ DHFR, uncompetitive interaction is observed for *E. coli* DHFR-TS^59^.

### Selective and dual inhibitors

The design of dual-acting inhibitors depends on the identification of compounds that have similar affinity for *Lc*PTR1 and *Lc*DHFR-TS, however one must bear in mind that these macromolecules have Km values that differ by more than 15 fold (substrate 25× (*Lc*PTR1 25 ± 2.1 µM vs. *Lc*DHFR-TS 1.0 ± 0.2 µM); cofactor 16× (*Lc*PTR1 37.2 ± 3.7 µM vs. *Lc*DHFR-TS 2.3 ± 0.5 µM)). In addition, IC_50_ values change according to substrate or enzyme concentration. For instance, when kinetic data is recorded at [S]=Km, the IC_50_/K*i* ratio for noncompetitive inhibitors is equal to 1/2. If higher concentrations of the substrate are employed, the ratio changes.

All kinetic data for *Lc*PTR1 was recorded at S/K_M_ ≅ 1.0, but the kinetic assays for *Lc*DHFR-TS were recorded at S/K_M_ = 5.0 for the substrate (S/K_M_=2.0 for the cofactor). Under these circumstances, the direct comparison of IC_50_ values is misleading. In order to circumvent this dilemma, the affinity of the compounds to their targets was assessed by their dissociation constant (*K_i_*), calculated with the Cheng–Prussov equation for competitive inhibitors^33^ (Table 1).

**Table 1.** Selectivity ratio for the 2,4-diaminopyrimidine derivates active against *Lc*DHFR-TS and *Lc*PTR1.

Compounds	*Lc*DHFR-TS		*Lc*PTR1		Selectivity *K*_i_*^Lc^*^PTR1^/ *K*_i_*^Lc^*^DHFR-TS^
	IC_50_ (µM)	*K*_i_ (µM)^a^	IC_50_ (µM)	*K*_i_ (µM)^a^	
**1**	1.65	0.28	3.00	1.50	5.45
**2**	18.00	3.00	4.10	2.05	0.68
**3**	4.20	0.70	4.60	2.30	3.29
**5**	>50^b^	>8.33^b^	0.94	0.47	<0.05

^a^Obtained from Cheng-Prussov equation^50^.

^b^Data from single-concentration assay.

This approach shows that compounds **1** and **3** have moderate selectivity for *Lc*DHFR-TS (*K_i_^Lc^*^PTR1^/*K_i_^Lc^*^DHFR-TS^ ratio  = 5.4 and 3.3-fold selectivity, respectively), whereas compound **2** has similar affinity to both targets (*K_i_^Lc^*^PTR1^/*K_i_^Lc^*^DHFR-TS^ ratio  = 0.68). Two out of three 2,4-diaminopyrimidine derivatives described are active against *Lm*DHFR-TS and *Lm*PTR1 have selectivity profile similar to compounds **1** and **3**: 6 to 13-fold selectivity for *Lm*DHFR-TS over *Lm*PTR1^24^. On the other hand, compound **5** is highly selective for *Lc*PTR1.

Despite the promising results presented so far, ADMET liabilities, such as high toxicity, might pose a severe hurdle to their advance as lead compounds. In order to evaluate this potential limitation, their predictive cardiac toxicity (hERG receptor inhibition model), mutagenicity (AMES test model) and carcinogenicity (Three-class model) was evaluated through admetSAR server (http://lmmd.ecust.edu.cn/admetsar1). These *in silico* models underscore no red light alerts and suggests oral acute toxicity doses higher than 500 mg/Kg for in vivo evaluation of all compounds (Figure S6).

## Conclusions

Although DHFR-TS and PTR1 from *L. major* have long been explored for drug development purposes, the same cannot be said about their counterparts in *L. chagasi*. This work sheds some light on this subject by reporting not only the optimal conditions to obtain *Lc*DHFR-TS and *Lc*PTR1, but also the balanced conditions for *in vitro* screening. This assay shows that 2,4 diaminopyrimidine derivatives, substituted at position 6, are competitive inhibitors of both enzymes whereas the 2,4-diaminoquinazoline derivative **5** is a selective inhibitor of *Lc*PTR1. This information shall guide the development of potent inhibitors with dissimilar selectivity profiles.

## Supplementary Material

Supplemental Material
