# Supplementary material for: Dual and selective inhibitors of pteridine reductase 1 (PTR1) and dihydrofolate reductase-thymidylate synthase (DHFR-TS) from Leishmania chagasi
Source: J Enzyme Inhib Med Chem. 2019 Aug 14;34(1):1439–50. doi: 10.1080/14756366.2019.1651311 (PMC6713189; doi:10.1080/14756366.2019.1651311)
Supplement: Supplemental Material [file IENZ_A_1651311_SM6895.docx]

**Dual and selective inhibitors of pteridine reductase 1 (PTR1) and dihydrofolate reductase-thymidylate synthase (DHFR-TS) from *Leishmania chagasi***

Bárbara Velame Ferreira Teixeira^a*^, André Lacerda Braga Teles^b,c,d*^, Suellen Gonçalves da Silva^a^, Camila Carane Bitencourt Brito^b^, Humberto Fonseca de Freitas^a,b^ , Acássia Benjamim Leal Pires^d^, Thamires Quadros Froes^b^ and Marcelo Santos Castilho^a,b^

^a^Programa de Pós-Graduação em Farmácia, Universidade Federal da Bahia, Salvador, BA, Brazil; ^b^Programa de Pós-Graduação em Biotecnologia, Universidade Estadual de Feira de Santana, Feira de Santana, BA, Brazil; ^c^Programa de Pós-Graduação em Ciências Farmacêuticas, Universidade Estadual da Bahia, Salvador, BA, Brazil; ^d^Departamento de Ciências da Vida, Universidade do Estado da Bahia, Salvador, BA, Brazil.

* Both authors contributed equally to this work.

Supplementary material


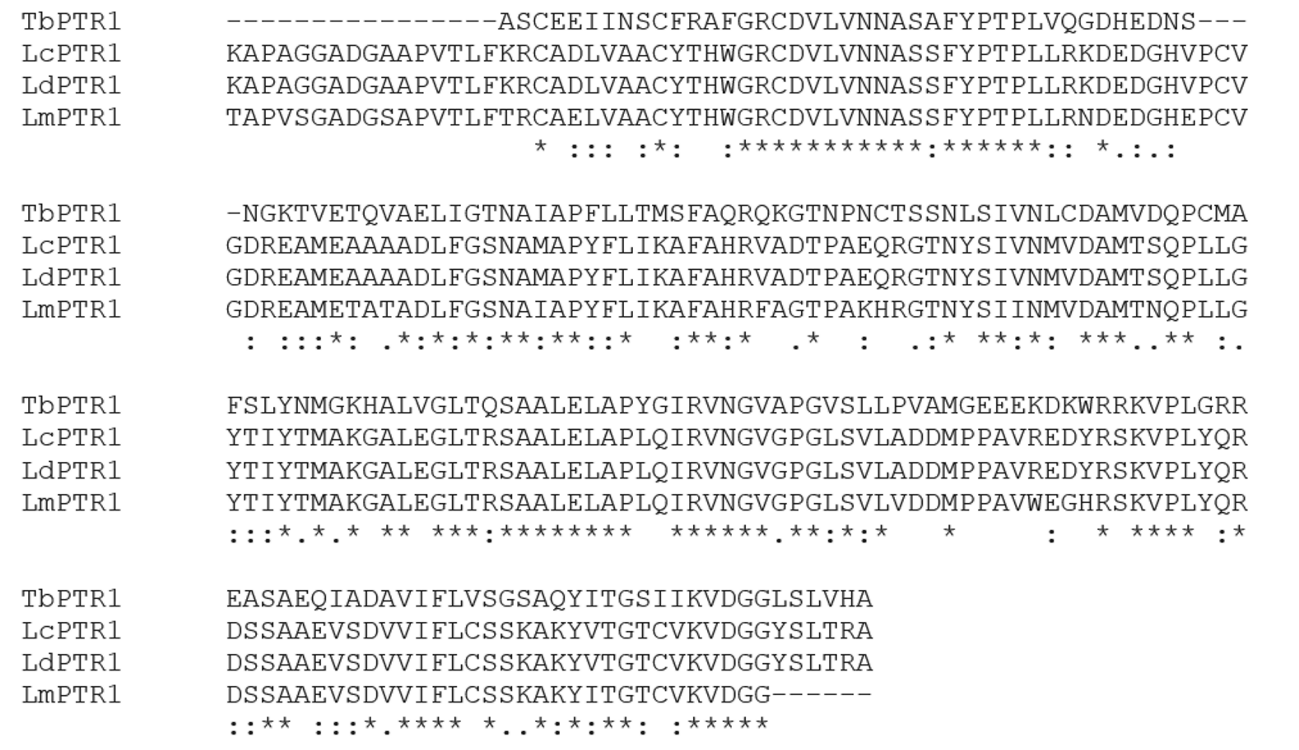


Figure S1. Sequential alignment of the *L. chagasi, L. donovani, L. major and T. Brucei* PTR1 generated on Clustal Omega server (https://www.ebi.ac.uk/Tools/msa/clustalo/). Overall sequential identity: *Lc*PTR1 x *Ld*PTR1 = 100%; *Lc*PTR1 x *Lm*PTR1 = 90%; *Lc*PTR1 x *Tb*PTR1 = 48.8%. ("*" = Identical, ":" = Similar, "." = Not conserved, "-" = absent).


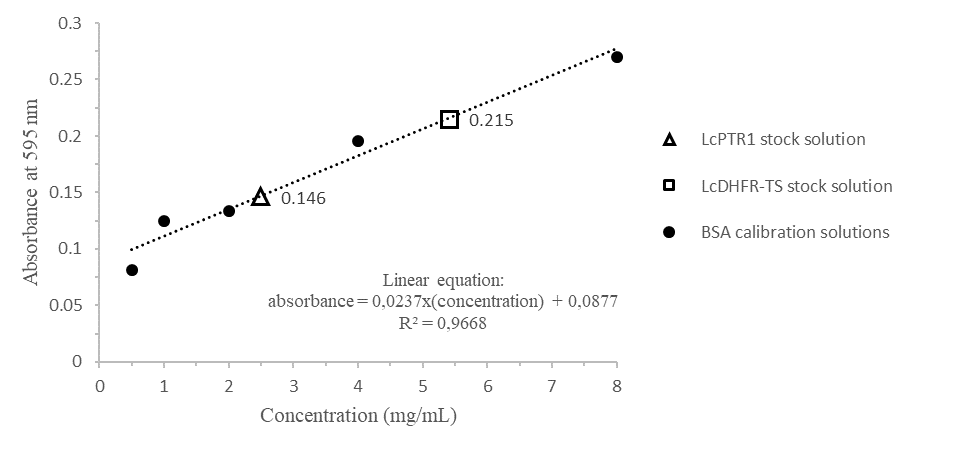


Figure S2. Calibration curve employed for protein concentration calculation with Bradford reagent. The absorbances of the *Lc*PTR1 and *Lc*DHFR-TS stock solutions are presented in the graph and correspond to the concentrations of 2.5 and 5.4 mg/mL, respectively.


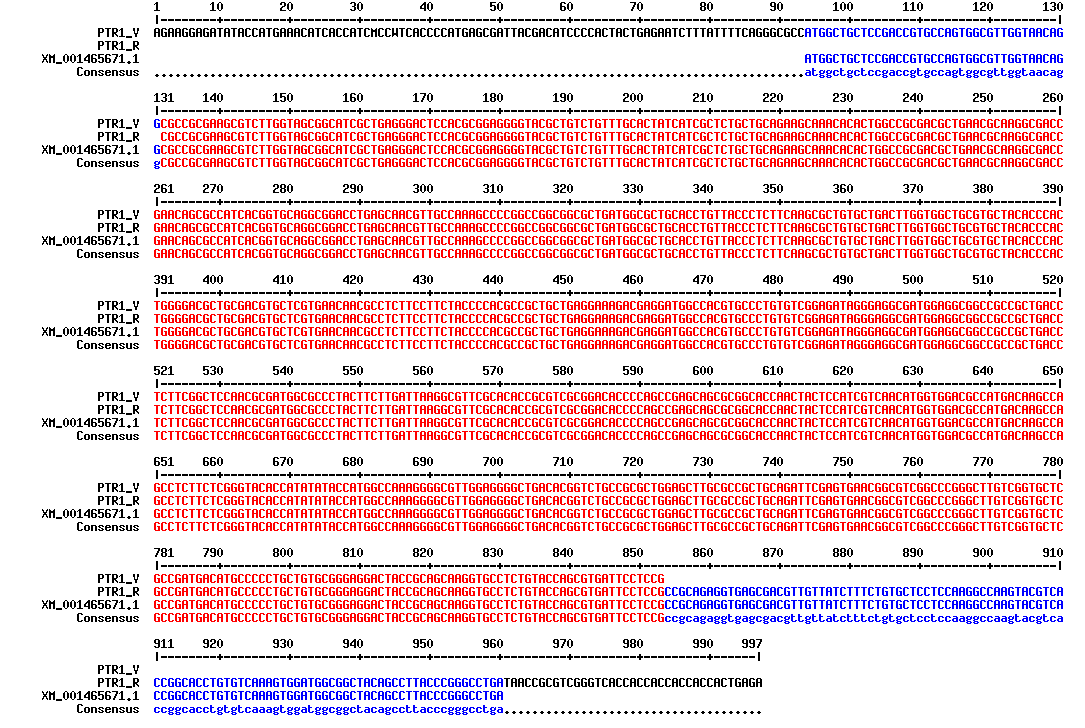


Figure S3. Sequence alignment between the product of the *Lc*PTR1 gene on pETM11 vector and its Genebank sequence (XM_001465671.1). PTR1_V: Forward sequencing; PTR1_R: Reverse sequencing; Consensus: lowercase letter: consensus between 2 sequences; capital letter: consensus among 3 sequences.


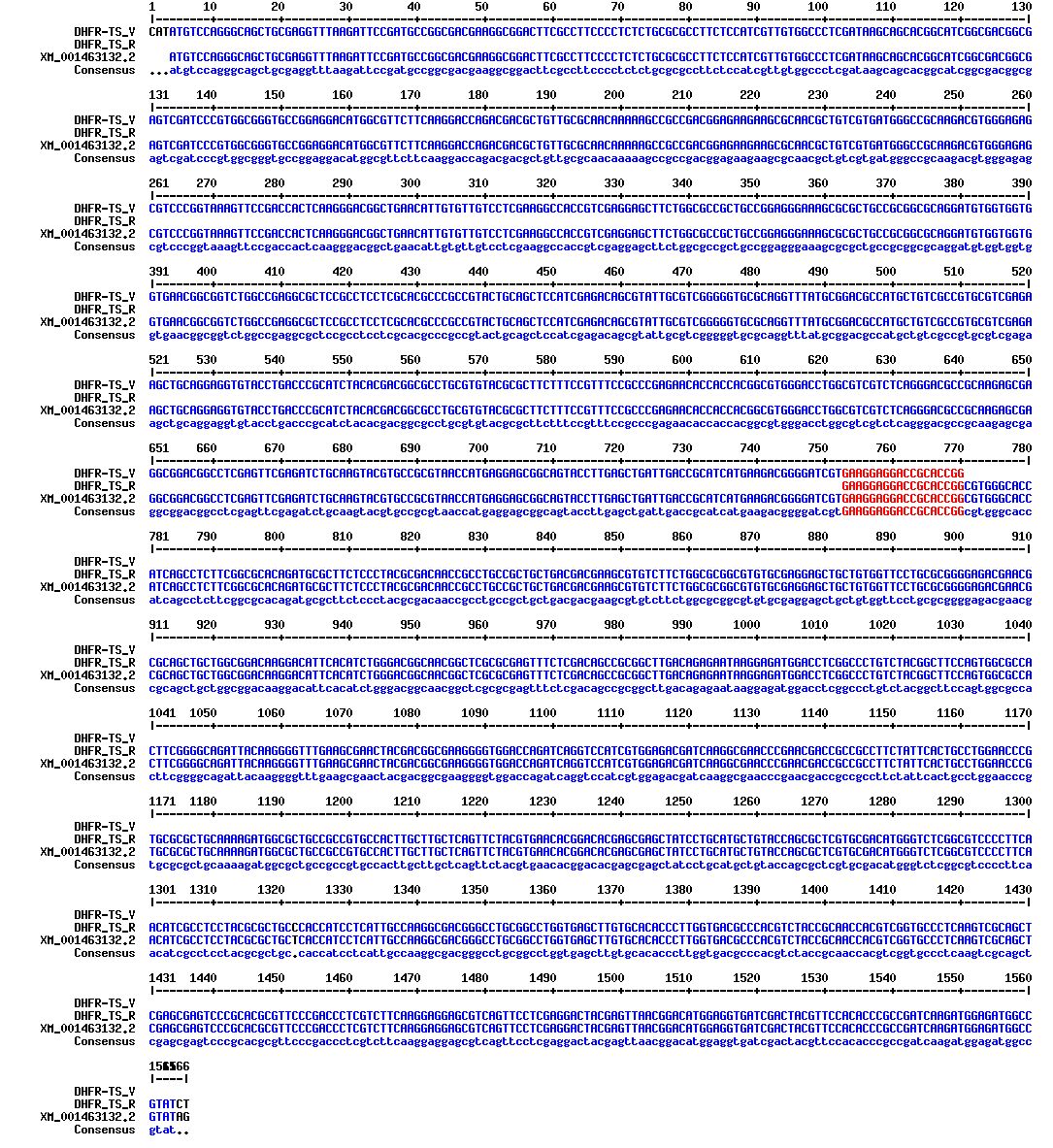


Figure S4. Sequence alignment between the product of the *Lc*DHFR-TS gene cloned on pET28a vector and its Genebank sequence (XM_001463132.2). DHFR-TS_V: Forward sequencing; DHFR-TS_R: Reverse sequencing; Consensus: lowercase letter: consensus between 2 sequences; capital letter: consensus among 3 sequences.


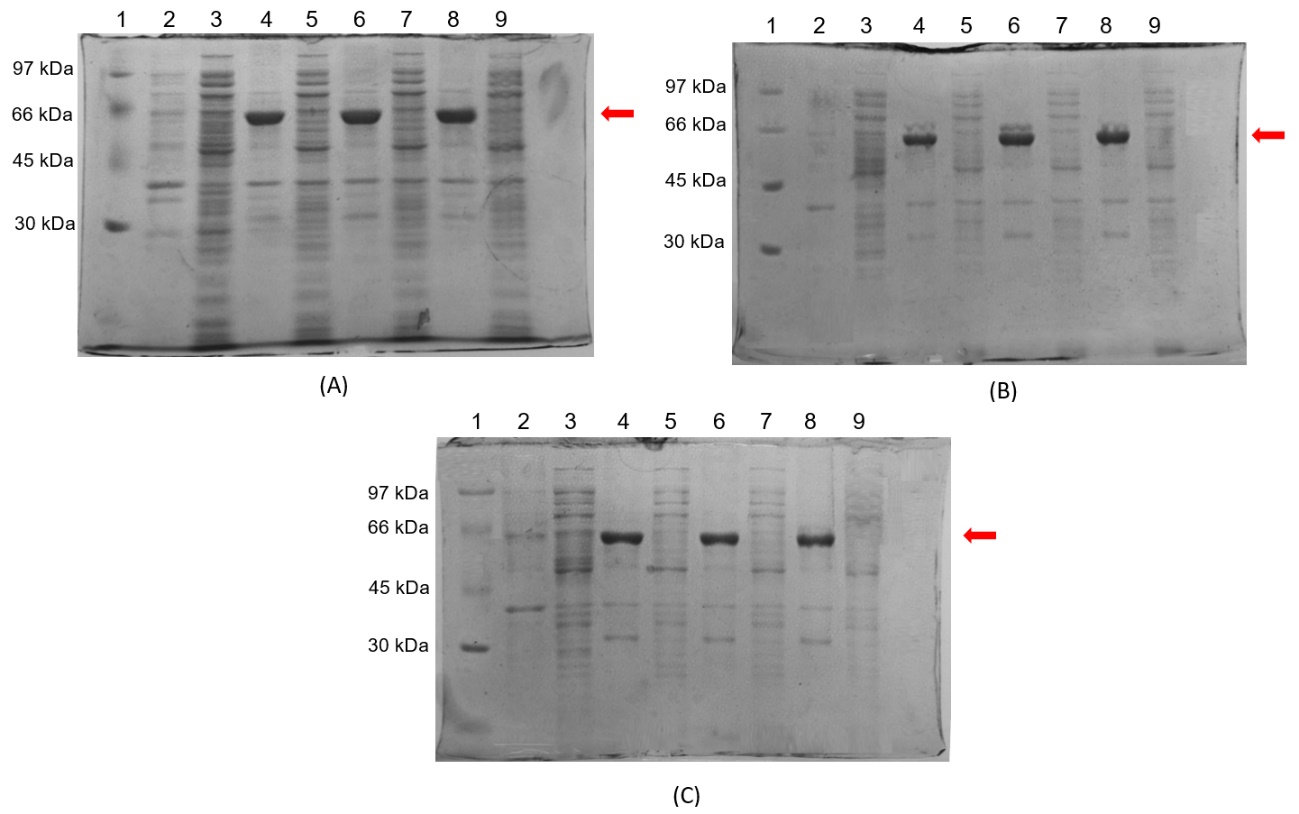


Figure S5. Screening of *Lc*DHFR-TS expression conditions in Bl21 (DE3). A) 18 °C for 24 hours; B) 25 °C for 16 hours; C) 37 °C for 24 hours; 1) LMW-SDS GE molecular weight standard; 2) Pellet for IPTG 0.0 mM; 3) Supernatant for IPTG 0.0 mM; 4) Pellet for IPTG 0.1 mM; 5) Supernatant for IPTG 0.1 mM; 6) Pellet for IPTG 0.5 mM; 7) Supernatant for IPTG 0.5 mM; 8) Pellet for IPTG 1.0 mM; 9) Supernatant for IPTG 1.0 mM. Red arrows indicate expected height for the *Lc*DHFR-TS band.

| **Toxicity Model** | **COMPOUND 1** | | **COMPOUND 2** | | **COMPOUND 3** | | **COMPOUND 4** | | **COMPOUND 5** | |
| --- | --- | --- | --- | --- | --- | --- | --- | --- | --- | --- |
|  | **Result** | **Probability** | **Result** | **Probability** | **Result** | **Probability** | **Result** | **Probability** | **Result** | **Probability** |
| **Human Ether-a-go-go-Related Gene Inhibition** | Weak inhibitor | 0.7577 | Weak inhibitor | 0.7577 | Weak inhibitor | 0.7577 | Weak inhibitor | 0.9724 | Weak inhibitor | 0.9436 |
|  | Non-inhibitor | 0.6832 | Non-inhibitor | 0.6832 | Non-inhibitor | 0.6832 | Non-inhibitor | 0.7159 | Non-inhibitor | 0.8210 |
| **AMES Toxicity** | Non AMES toxic | 0.7062 | Non AMES toxic | 0.7062 | Non AMES toxic | 0.7062 | Non AMES toxic | 0.7905 | Non AMES toxic | 0.7086 |
| **Carcinogens** | Non-carcinogens | 0.9538 | Non-carcinogens | 0.9538 | Non-carcinogens | 0.9538 | Non-carcinogens | 0.8606 | Non-carcinogens | 0.9282 |
| **Biodegradation** | Not ready biodegradable | 0.9779 | Not ready biodegradable | 0.9779 | Not ready biodegradable | 0.9779 | Not ready biodegradable | 0.9962 | Not ready biodegradable | 0.9829 |
| **Acute Oral Toxicity** | Dose > 500 mg/Kg | 0.4953 | Dose > 500 mg/Kg | 0.7577 | Dose > 500 mg/Kg | 0.4953 | Dose > 500 mg/Kg | 0.5171 | Dose > 500 mg/Kg | 0.6909 |

Figure S6. Toxicity prediction for the 2,4-diaminopyrimidine derivatives employed in this work, according to admetSAR server (http://lmmd.ecust.edu.cn/admetsar1).
